# Plant Chemistry and Local Adaptation of a Specialized Folivore

**DOI:** 10.1371/journal.pone.0038225

**Published:** 2012-05-30

**Authors:** Liisa Laukkanen, Roosa Leimu, Anne Muola, Marianna Lilley, Juha-Pekka Salminen, Pia Mutikainen

**Affiliations:** 1 Department of Biology, Section of Ecology, University of Turku, Turku, Finland; 2 Department of Plant Sciences, University of Oxford, Oxford, United Kingdom; 3 Department of Chemistry, University of Turku, Turku, Finland; 4 Institute of Integrative Biology, ETH-Zürich, ETH-Zentrum, Zürich, Switzerland; University of Helsinki, Finland

## Abstract

Local adaptation is central for creating and maintaining spatial variation in plant-herbivore interactions. Short-lived insect herbivores feeding on long-lived plants are likely to adapt to their local host plants, because of their short generation time, poor dispersal, and geographically varying selection due to variation in plant defences. In a reciprocal feeding trial, we investigated the impact of geographic variation in plant secondary chemistry of a long-lived plant, *Vincetoxicum hirundinaria*, on among-population variation in local adaptation of a specialist leaf-feeding herbivore, *Abrostola asclepiadis*. The occurrence and degree of local adaptation varied among populations. This variation correlated with qualitative and quantitative differences in plant chemistry among the plant populations. These findings provide insights into the mechanisms driving variation in local adaptation in this specialized plant-herbivore interaction.

## Introduction

Local adaptation is central for creating and maintaining spatial variation in antagonistic interactions [1]–[3]. Spatial variation in fitness-related traits of the interacting species, such as host defence and counter-defence of the enemy, may reflect divergent selection among populations, which often results in local adaptation [2], [4]. Natural enemies are locally adapted if their fitness is higher on hosts from sympatric (i.e. home) population compared to those from allopatric (i.e. away) populations. In interactions with a long-lived host and an enemy with much shorter generation time, the enemy is predicted to be locally adapted to its sympatric host [5], [6]. Furthermore, adaptation to local host populations is likely to evolve when the enemy has strong negative effects on host fitness and when the migration rate of the enemy is higher than that of the host [7], [8]. Several studies including plant-herbivore, plant-pathogen, and animal systems have observed local adaptation of natural enemies to their hosts (e.g., [4], [9], [10]).

In general, local adaptation is predicted to be more likely among strongly differentiated populations that are located in clearly diverged environments [11]–[13]. However, local adaptation can occur even within continuous populations or between connected populations, if gene flow among patches or populations is not strong enough to counteract the forces of selection [2], [14]. Furthermore, the occurrence and degree of local adaptation in antagonistic interactions is predicted to vary among populations and in time due to the dynamic nature of the evolutionary process [8], [15], [16]. At a given point in time, populations of hosts and enemies might show different degrees of local adaptation, or even lack of local adaptation, depending on the strength of the selection imposed by the interacting species [4], [8]. This variation is predicted to be driven by differences in the traits that are central for the interaction, such as host resistance and tolerance [4].

In accordance with these predictions, some studies on local adaptation in plant-herbivore interactions demonstrate that herbivores are locally adapted (e.g., [9], [17]) while other studies provide no evidence on local adaptation (e.g., [18], [19]). Moreover, the occurrence and degree of local adaptation of insect herbivores to their sympatric host plant populations is often found to vary among populations (e.g., [20], [21]). Geographic variation in plant resistance traits, such as secondary chemistry or structural defence, may drive spatial variation in local adaptation of specialist herbivores with short generation times [17]. Plant resistance forms a strong selection pressure that acts upon the performance of insect herbivores [22]. Although it has been shown that between-species variation in defence chemistry can influence herbivore adaptation to different host plant species [23], our study is among the first ones to investigate how qualitative and quantitative differences in host-plant secondary chemicals within a species drive variation in local adaptation among natural populations of a specialist herbivore. Given that human activities are rapidly changing our biological landscapes and consequently altering evolutionary trajectories, it is becoming increasingly important to understand the factors affecting local adaptation and evolution of interactions among species.

We investigated local adaptation of the specialist herbivorous moth, *Abrostola asclepiadis* Schiff. (Lepidoptera), on its host plant, *Vincetoxicum hirundinaria* Med. ( = *Cynanchum vincetoxicum* (L.) Pers.) (Apocynaceae, former: Asclepiadaceae), in a reciprocal feeding trial using three populations located in the southwestern archipelago of Finland. The host plant populations are genetically differentiated [24], which indicates restricted gene flow between populations. This can enhance local adaptation of herbivores, if gene flow between herbivore populations is greater than that between plant populations [7], [8]. Our previous investigations have also demonstrated that spatial variation in the associations of host plant chemistry, levels of damage by the herbivore, and plant fitness reflect a selection mosaic (sensu [15], [25]). Moreover, we have also demonstrated that the associations of plant chemicals and herbivore damage by *A. asclepiadis* vary among the populations and range from negative to positive [25]. Therefore, a given chemical may be positively associated with the level of herbivory in one population, that is the chemical compound functions as an attractant for specialist herbivores, and negatively in an another population indicating a function as herbivore defence [25]. In this study, we were interested in whether local adaptation of *A. asclepiadis* varies among populations, and whether this variation is influenced by among-population variation in secondary chemistry of its host plant, *V. hirundinaria*. To answer these questions, we conducted a resiprocal feeding trial in the laboratory with both plants and larvae from the three sites.

## Methods

### Study species and populations

*Vincetoxicum hirundinaria* Med. ( = *Cynanchum vincetoxicum* (L.) Pers.) (Apocynaceae, former: Asclepiadaceae) is a long-lived perennial herb with a life span of several decades [R. Leimu, unpublished data]. It typically grows on exposed slopes and cliffs and prefers calcareous substrates and has a wide continental Eurasian distribution. The north-western limit of the distribution is in Scandinavia, where *V. hirundinaria* inhabits the islands and coastal areas of the Baltic Sea. Population sizes in this area range from tens to thousands of individuals and distances among the populations vary from less than one to tens of kilometres.

*Vincetoxicum hirundinaria* is highly poisonous and contains several types of secondary compounds, such as antofine and phenolic compounds [25], which might explain the low number of herbivores feeding on it. In our study area only three specialized herbivores, *Lygaeus equestris* L. (Heteroptera), *Euphranta connexa* (Fabr.) (Diptera), and *Abrostola asclepiadis* Schiff. (Lepidoptera) feed on *V. hirundinaria*. Here we focused on the folivorous noctuid moth *A. asclepiadis* that is a strict specialist on *V. hirundinaria*. It can be locally common, and its population sizes vary both spatially and between years ([26] L. Laukkanen, personal observation). Consequently, the damage levels also vary among years and populations from no damage to almost complete defoliation [25]–[27]. The female moth oviposits on the leaves of *V. hirundinaria* in June and July. In the field, eggs hatch approximately ten days after oviposition and the five larval instars are completed in about five to six weeks [26]. In the laboratory, the development is faster (see below). *Abrostola asclepiadis* can disperse up to 50 km under optimal conditions [26].

We used *V. hirundinaria* plants and larvae of *A. asclepiadis* from three island populations located in the southwestern archipelago of Finland: Anskär (N 60°11.6′, E 21°41.9′), Jurmo (N 59°49.5′, E 21°34.8′), and Lammasluoto (N 60°14.0′, E 21°56.8′). The abundance of *A. asclepiadis* was the most important factor contributing to the selection of the study populations, as we wanted to ensure sufficient number of eggs for the experiment. The selected populations form a transect from the inner to the outer archipelago and thus represent the distribution of *V. hirundinaria* in the study area. The distances among these populations vary from 15.6 to 49.6 km and the distances to the nearest *V. hirundinaria* population vary from 0.7 to 4.7 km. *Vincetoxicum hirundinaria* is abundant in the three selected populations: the plant population sizes vary from about 2 500 to 5 000 individuals ([28] L. Laukkanen and A. Muola, personal observations).

All necessary permits were obtained for the described field studies. Forest administration of Finland (Metsähallitus) granted a licence to collect *V. hirundinaria* and *L. equestris* from the area of Archipelago national park. Our studies did not involve endangered or protected species.

### Experimental design

To investigate if *A. asclepiadis* is adapted to its local host plant populations, we designed a reciprocal feeding trial in the laboratory with both plants and larvae from the three sites (Anskär, Jurmo, Lammasluoto). We haphazardly selected plants from the three populations, dug up rootstocks of 33 plant individuals (11 plants per population) and potted the plants at the end of May 2006. The plants were grown in a greenhouse for six weeks before the experiment. The use of adult plants rather than individuals grown from seeds means that, in addition to genetic differences, the study plants may differ due to environmental effects the plants have experienced in their populations of origin. Therefore, all of the variation in plant quality is not likely to be genetically based or lead to evolutionary changes. On the other hand, the study plants represent the natural situation that the herbivores meet in each of the habitats and have to adapt to.

We collected egg clusters of *A. asclepiadis* from the same three populations (18–20 clusters per population) in late June–July 2006. The eggs were kept at room temperature (20°C) and in natural light conditions before hatching.

In the reciprocal feeding trial larvae from each of the three sites were fed on plants originating from each of the three sites. We used 31 plant individuals (9–11 plants per population) in the experiment. Some of the eggs were infertile or parasitized and, thus, we used 6–13 egg clusters per population (1–8 eggs per cluster) in the experiment. We had 11.3±1.86 (mean ± se) replicates for each of combination of plant population by herbivore population, and the total number of larvae was 102. Each larva was randomly assigned for a particular plant immediately after hatching in July and later on fed with fresh leaves collected from this particular plant. To control for the effect of genetic differences among the egg clusters, the larvae from each egg cluster were divided among the three plant populations. Furthermore, larvae from the three populations were assigned to feed on each plant individual to control for the effect of differences among plant individuals. We reared the larvae individually in plastic vials for the whole larval period from the first instar to the pupal stage (about 15 days). The vials were kept under natural light conditions and at room temperature (20°C). We replaced the leaves with fresh ones every second day during the first week and later on every day until the larval stage was finished. We fed each larva with leaves from one plant individual during the whole larval period. At the end of the larval period we weighed the larvae to obtain larval biomass and recorded the developmental time of the larvae (days from hatching to pupation). After the pupation, we weighed the pupae and determined the sex of each individual. We also recorded the survival of the larvae until pupation.

### Chemical analysis

To analyze leaf secondary chemistry we collected leaf samples from all plants used in the experiment. We collected two randomly selected average-size mid-stem leaves from each plant individual at the third day of the experiment and placed the leaves into sealed plastic vials that were immediately frozen (−18°C). The leaf samples were freeze dried six months after the collection, homogenized, and stored in the freezer until the chemical analyses were conducted. The contents of leaf compounds were analyzed with high-performance liquid chromatography (HPLC) assisted with diode-array detection (for detailed methods see [23]). Here we focus on the contents of total lipophilic compounds (25 different lipophilic compounds analyzed), total flavonoids (13 different flavonoids analyzed), chlorogenic acid, catechin derivatives, and antofine that are likely to be important in the interaction of *V. hirundinaria* and its herbivores [25], [29]. Lipophilic compounds form a relatively large group of chemical compounds including, for instance, chlorophylls and carotenoids. Phenolic compounds (flavonoids, chlorogenic acid, and catechin derivatives) have many ecological and physiological roles in plants and they have been shown to be important for plant–herbivore interactions (e.g., [30], [31]). Antofine, a phenanthroindolizidine alkaloid, is known for its cytotoxic activity [32]. The contents of all measured compounds are presented as mg/g dry weight. In this study, variation in leaf chemistry among plant individuals is unlikely to be caused by the removal of leaves for feeding the larvae, as the resistance of *V. hirundinaria* to *A. asclepiadis* is not inducible by defoliation [A. Muola, unpublished data].

### Statistical analysis

To investigate if the measured fitness-related traits were correlated, we tested for correlations between larval biomass, pupal mass and developmental time by calculating Pearson's correlation coefficients between these traits. Larval biomass correlated positively and strongly with pupal mass (*A. asclepiadis* on sympatric plants: r = 0.906, n = 31, *p*<0.001). Larval developmental time correlated negatively with pupal mass, i.e., the larvae that developed faster were heavier (*A. asclepiadis* on sympatric plants: r = −0.436, n = 31, *p* = 0.014). Because of the significant correlations between the fitness related traits, we only included pupal mass and survival in the analyses of local adaptation. Pupal mass has also been shown to correlate with fitness in other lepidopteran herbivores [33]–[35].

We used logistic regression to investigate differences in survival among the herbivore populations and host plant populations. Plant population, herbivore population, and their interaction were used as factors in the analysis. A significant interaction between plant population and herbivore population indicates local adaptation of the herbivore if survival is higher on plants from the sympatric plant population compared to that on plants from allopatric populations. Sex was not included in this model, because it could not be determined at larval stage. Given the lack of local adaptation and low variation in survival rates (see results) no further analyses on the impact of plant chemistry on survival were conducted.

We used analysis of covariance (ANCOVA) to investigate among-population variation in pupal mass of *A. asclepiadis* and whether this variation is associated with variation in leaf chemistry. Plant population, herbivore population, herbivore sex, and their interactions were used as factors and the contents of five chemical components (total lipophilic compounds, total flavonoids, chlorogenic acid, catechin derivatives, and antofine) were included as covariates. Sex was included in the model to account for differences in size between male and female pupae [36], [37]. A significant interaction between plant population and herbivore population indicates local adaptation of the herbivores, if herbivore performance is higher on plants from the sympatric plant population compared to that on plants from allopatric populations. This is the home - away comparison presented by Kawecki and Ebert [2]. Significant interactions between the covariates and plant and/or herbivore population indicate that the effect of the contents of the chemical compounds on herbivore performance varies among plant and/or herbivore populations. We simplified the model by removing non-significant covariates and their interactions with the factors starting from the complete model that included all main factors, all covariates, and all of their interactions (backward elimination). However, we did not remove non-significant interactions that the experiment was designed to test for, namely the interactions between sex, plant population, and herbivore population. (We also conducted the ANCOVA-model with all interactions included, see Table S1.) Please note, that in some cases degrees of freedom seem to differ from expected, which is due to the complex covariance structures that affect the degrees of freedom [38].

Given the statistically significant interaction between herbivore population and plant population (see results), we further tested for local adaptation in the whole dataset and for each of the three herbivore populations separately by comparing the pupal mass of *A. asclepiadis* between sympatric vs. allopatric host plants. For the whole data set, we used a two-way ANOVA with plant population, herbivore population, and their two-way interaction as factors and constructed a contrast for the interaction between the host population and herbivore population to test the difference in pupal mass of *A. asclepiadis* between sympatric vs. allopatric host plants. For each of the herbivore populations, we ran ANOVAs with a Helmert contrast comparing the sympatric and the two allopatric populations. For Anskär and Jurmo herbivore populations we used ANCOVAs where plant population, herbivore sex, and their two-way interaction were included as factors and the contents of five chemical components as covariates. We simplified also these models by removing non-significant covariates and their interactions with the factors. The covariates (i.e. the chemical compounds) had to be exluded from the model of Lammasluoto because of the low number of replicates. Therefore, only plant population, herbivore sex, and their two-way interaction were included in analyses of the data on Lammasluoto.

We also tested for among-population variation in the contents of the five plant chemical compounds (total lipophilic compounds, total flavonoids, chlorogenic acid, catechin derivatives, and antofine) with multivariate analysis of variance (MANOVA). We used Tukey's HSD test for pairwise differences in the contents of the chemical compounds between the populations.

Outliers in the data were detected using Grubb's test [39]. Based on the results, one observation was excluded from the data on antofine content. Multivariate analysis of variance was conducted using the the SAS statistical package (version SAS 9.2) (SAS Institute Inc., 2002–2007). Other statistical analysis were conducted with SPSS (SPSS Inc. 2009).

## Results

### Among-population variation in herbivore local adaptation

Overall, 91% of the larvae survived and pupated in the experiment. There were no significant differences in survival among the herbivore populations (Wald = 4.83, df = 2, *p* = 0.785) or among the host plant populations (Wald = 0.275, df = 2, *p* = 0.872; herbivore by host plant population interaction Wald = 0.022, df = 4, *p* = 0.999). These results suggest lack of local adaptation: survival did not differ between herbivores fed on sympatric and allopatric host plants. Therefore, the data on survival were not analysed further.

**Figure 1:**
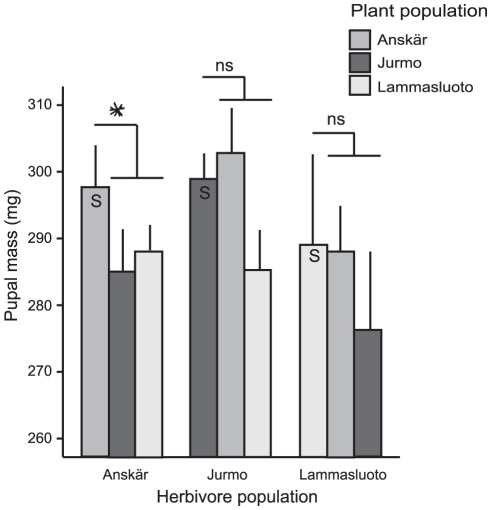
Mean (± S.E.) pupal mass of *Abrostola asclepiadis* in a reciprocal feeding trial. In a reciprocal feeding trial, *A. asclepiadis* larvae from three sites (Herbivore population) were grown on plants from the same three sites (Plant population). S denotes sympatric combinations of plant and herbivore populations. The asterisk and “ns” indicate statistical significance (*P*<0.05 or *P*>0.05, respectively) of contrasts comparing pupal mass of herbivores feeding on sympatric host plants to that on allopatric host plants from the two other populations.

There was a statistically significant interaction between plant population and herbivore population for the pupal mass of *A. asclepiadis*, but the main effects of plant and herbivore population were both non-significant (Table 1, Fig. 1). The contrast comparing pupal mass of *A. asclepiadis* between sympatric and allopatric host plants for the whole dataset was non-significant (F_1,73_ = 1.727, *p* = 0.193). The average pupal mass of *A. asclepiadis* was 297.05±3.53 g (mean ± se) on sympatric hosts and 289.8±3.01 g on allopatric hosts, which is a 2.5% difference in pupal mass.

**Table 1:** Results of ANCOVA on the effects of plant and herbivore population of origin, herbivore sex, and leaf chemistry on pupal mass of *Abrostola asclepiadis*.

Source of variation	df	F	*p*
Plant population	2	2.77	0.074
Herbivore population	2	1.62	0.210
Sex	1	2.71	0.107
Plant population×Herbivore population	2	4.15	0.023
Plant population×Sex	2	0.74	0.482
Herbivore population×Sex	2	2.45	0.098
Plant population×Herbivore population×Sex	2	0.21	0.808
Lipophilic compounds	1	2.34	0.134
Flavonoids	1	3.24	0.079
Chlorogenic acid	1	3.80	0.058
Catechin derivatives	1	0.89	0.350
Antofine	1	2.63	0.112
Plant population×Lipophilic compounds	2	4.05	0.025
Plant population×Flavonoids	2	0.27	0.763
Herbivore population×Lipophilic compounds	1	0.07	0.793
Herbivore population×Flavonoids	1	0.07	0.788
Herbivore population×Chlorogenic acid	1	5.73	0.021
Plant popul.×Herbivore popul.×Lipophilic compounds	2	3.42	0.042
Plant popul.×Herbivore popul.×Flavonoids	2	3.19	0.051
Error	42		

We had three herbivore and three plant populations in a reciprocal feeding trial.

### Effect of host plant chemistry on herbivore local adaptation

**Figure 2:**
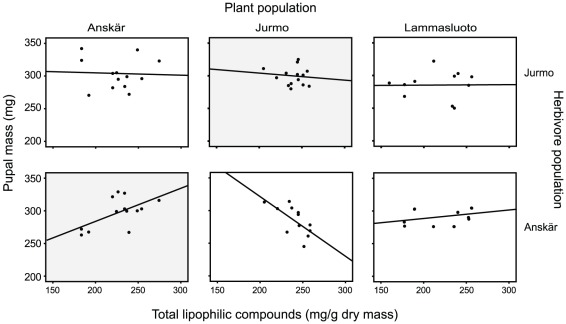
Effect of total lipophilic compounds on pupal mass of *Abrostola asclepiadis* larvae. In a reciprocal feeding trial, *A. asclepiadis* larvae from three sites (Herbivore population) were grown on plants from the same three sites (Plant population). The sympatric combinations of plant and herbivore populations are marked with gray background. Due to low number of replication in one of the populations, we present the data only for two of the herbivore populations.

Host plant chemistry seemed to modify the variation in herbivore pupal mass among the plant and/or herbivore populations as indicated by three significant interactions between plant population, herbivore population and the investigated chemical compounds (Table 1). First, the three-way interaction between plant population, herbivore population, and total lipophilic compounds was significant, suggesting that the effect of total lipophilic compounds on pupal mass of the herbivores varied among the herbivore populations and also depended on the origin of the host plant (*p* = 0.042; Table 1). Given the significant interaction, we tested for local adaptation and the effects of chemical compounds on variation in pupal mass separately for each of the herbivore populations. The larvae from Anskär performed better on their sympatric host plants compared to allopatric ones; the contrast comparing pupal mass on sympatric host plants to that on allopatric host plants from two populations is significant suggesting local adaptation of the herbivores (*p* = 0.039). The effect of increasing content of lipophilic compounds on the pupal mass of larvae originating from Anskär differed among the plant populations (plant population by lipophilic compounds: F_2,20_ = 6.363, *p* = 0.007; Fig. 2). When feeding on sympatric host plants, the pupal mass of larvae from Anskär correlated positively with the content of lipophilic compounds. A weak positive relationship was also observed for larvae from Anskär feeding on host plants from the Lammasluoto population. Unlike on the two other plant populations, the pupal mass of larvae from Anskär correlated negatively with the content of lipophilic compounds when feeding on plants from Jurmo (Fig. 2). For the herbivores from Jurmo, the contrast comparing pupal mass on sympatric host plants to that on allopatric host plants from two populations was not significant (*p* = 0.709). Furthermore, the pupal mass of herbivores from Jurmo was not significantly related to the content of total lipophilic compounds regardless of plant population (plant population by lipophilic compounds: non-significant and thus removed from the final model; main effect of lipophilic compounds: F_1,26_ = 2.882, *p* = 0.102; Fig. 2). For the herbivores from Lammasluoto, the contrast comparing pupal mass on sympatric host plants to that on allopatric host plants from two populations was not significant (*p* = 0.522). In Lammasluoto we could not determine the statistical significance of variation in the content of lipophilic compounds, or other chemicals analyzed, on the pupal mass because of low degrees of freedom.

**Figure 3:**
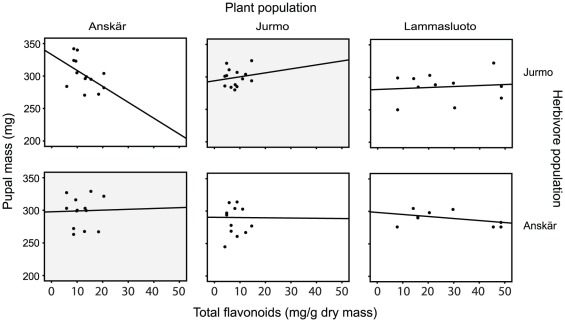
Effect of total flavonoids on pupal mass of *Abrostola asclepiadis* larvae. In a reciprocal feeding trial, *A. asclepiadis* larvae from three sites (Herbivore population) were grown on plants from the same three sites (Plant population). The sympatric combinations of plant and herbivore populations are marked with gray background. Due to low number of replication in one of the populations, we present the data only for two of the herbivore populations.

Secondly, the three-way interaction between plant population, herbivore population, and total flavonoids was statistically almost significant (*p* = 0.051; Table 1), suggesting that the effect of total flavonoids on pupal mass of the herbivore varied among the herbivore populations and also depended on the origin of the host plant. In contrast to the results on the lipophilic compounds, the pupal mass of herbivores from Anskär was not related to the content of total flavonoids regardless of plant population (plant population by flavonoids: non-significant and removed from the final model, main effect of flavonoids: F_1,20_ = 0.213, *p* = 0.649; Fig. 3). By contrast, the effect of increasing content of flavonoids on the pupal mass of larvae originating from Jurmo tended to differ among the plant populations indicated by the nearly significant interaction between plant population and flavonoid content (F_1,26_ = 3.276, *p* = 0.082; Fig. 3). For Jurmo, the pupal mass of larvae feeding on sympatric host plants correlated weakly positively with the flavonoid content. In contrast to the sympatric combination, larvae from Jurmo feeding on plants from Anskär showed a clear negative trend; the pupal mass was higher when the total flavonoids content was low (Fig. 3).

**Figure 4:**
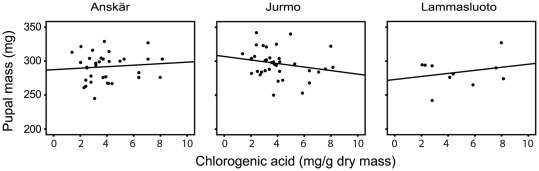
Effects of chlorogenic acid content on pupal mass of *Abrostola asclepiadis* from three populations.

Thirdly, we found a significant two-way interaction between herbivore population and the content of chlorogenic acid (*p* = 0.021; Table 1 and Figure 4) suggesting that the effect of chlorogenic acid on pupal mass of *A. asclepiadis* varied among the herbivore populations. The pupal mass of larvae from Anskär (F_1,20_ = 0.234, *p* = 0.634) and Lammasluoto correlated positively, with the content of chlorogenic acid, whereas in Jurmo the relationship was slightly negative and nearly significant; i.e. the higher the chlorogenic acid content, the lighter the larva (F_1,26_ = 3.295, *p* = 0.081; Fig. 4).

### Among-population variation in plant chemical compounds

We found significant phenotypic differences in secondary chemistry among the three plant populations (MANOVA, Pillai's trace F_10,48_ = 2.84, *p* = 0.007; Table 2). Lammasluoto population differed from the other two populations; the differences were statistically significant for concentrations of flavonoids and antofine (Table 2).

**Table 2:** Mean (± S.E.) concentrations of five chemical compounds in the leaves of *Vincetoxicum hirundinaria* from three populations.

Chemical compound	Anskär	Jurmo	Lammasl.	F	*p*
Lipophilic compounds	230±7.9	238±4.8	218±13.1	1.38	0.269
Flavonoids	12.5±1.3 a	8.5±1.0 a	23.7±4.5 b	10.68	<0.001
Chlorogenic acid	4.0±0.43	3.2±0.25	5.0±0.83	3.19	0.057
Catechin derivatives	0.82±0.13	0.61±0.07	1.2±0.27	3.24	0.055
Antofine	0.46±0.06 a	0.52±0.07 a	0.97±0.11 b	11.83	<0.001

All concentrations are presented as mg/g dry weight. Overall, the concentrations differ significantly among the populations (MANOVA, Pillai's trace F_10,48_ = 2.84, *p* = 0.007). The *F*- and *p*- values represent the results of one-way ANOVAs for differences among the populations for each compound separately. The letters indicate significant difference (*p*<0.05) in the amount of each chemical compound between plant populations.

## Discussion

Overall, in line with theoretical predictions, we found among-population variation in local adaptation of the specialist leaf-feeding herbivore, *Abrostola asclepiadis*, to the sympatric populations of its host plant, *Vincetoxicum hirundinaria*. The herbivores from one of the populations (Anskär) performed significantly better on their sympatric host plant population compared to allopatric host plant populations. Herbivores from the other two populations (Jurmo and Lammasluoto) were not locally adapted to their sympatric host plant populations. Spatial variation in local adaptation of herbivores to their host plant populations has been found in several studies (e.g., [20], [21]). Although a few studies demonstrate both the selective impact of insect herbivores on plant secondary chemistry and that plant chemistry can influence the abundance and performance of herbivores [40], [41], the selective factors that cause variation in local adaptation are not yet thoroughly explored. Our results suggest that variation in herbivore local adaptation may be driven by the qualitative and quantitative among-population divergence in host plant chemistry.

Theoretical models on the evolution of interactions between hosts and their natural enemies predict that local adaptation is more likely to occur if the enemies have a higher migration rate than their hosts [7]. In our study system, the level of genetic differentiation among the host plant populations is relatively low, though statistically significant (average F_ST_ = 0.052), suggesting moderate migration rates among the populations [24]. *Vincetoxicum hirundinaria* is insect pollinated, but also capable of self-fertilization [42], which might decrease migration rates among the populations and increase genetic differentiation of populations. On the other hand, the seeds are wind dispersed, but also capable of dispersing by floating on the water between the islands ([43] R. Leimu, unpublished data), which may promote longer-distance dispersal between the islands. As the adult *A. asclepiadis* may migrate tens of kilometres under optimal conditions [26], it seems probable that the herbivores have higher migration rates than the host plant, which fits the predictions of the model of Gandon et al. [7]. The generation time of *A. asclepiadis* is also considerably shorter than the generation time of *V. hirundinaria*, which, together with the high migration rate suggests that *A. asclepiadis* is likely to show local adaptation. Therefore, our results showing local adaptation are in line with predictions based on the characteristics of our study system.

### Variation in plant chemistry contributes to local adaptation

Plant defences and their effects on herbivores often vary substantially among plant populations due to genetic and environmental differences [44]–[46]. Such geographic variation in plant defences results in variation in selection pressures that the host plants exert on their herbivores. In an earlier study, we reported wide quantitative and qualitative variation in leaf chemistry among *V. hirundinaria* populations, and showed how this variation is strongly linked to spatial variation in the levels of herbivory and the selection the herbivores exert on their host plants in a selection mosaic [25]. Leaf chemistry also varied significantly among the three populations in the current study (Table 2). It was, therefore, reasonable to expect that variation in plant chemistry plays an important role in the variation in local adaptation of the herbivore *A. asclepiadis*.

In line with our prediction, variation in plant chemistry was linked to herbivore local adaptation. We found that an increase in total lipophilic compounds in plants from the sympatric host plant population increased the fitness of the herbivores from the locally adapted Anskär population. By contrast, when fed on plants from the two allopatric populations (Jurmo and Lammasluoto) the performance of the herbivores from Anskär was either negatively associated with increased lipophilic compounds (plants from Jurmo) or only weakly influenced by the lipophilic compounds (plants from Lammasluoto). This result reflects the qualitative variation in lipophilic compounds among the plant populations observed by Muola et al. [25], and the fact that herbivores from the Anskär population can tolerate or detoxify especially those lipophilic compounds that their sympatric host plants contain. Specialist herbivores may adapt to tolerate or detoxify the specific chemical compounds of their host plants, and may even be attracted by high levels of certain defensive compounds [47]–[51]. However, it is likely that this ability to process certain plant chemicals varies among populations of herbivores, and that herbivores may be able to detoxify especially those chemicals that their sympatric host plants contain. As a specialist herbivore, *A. asclepiadis* is likely to adapt to the specific lipophilic compounds of the sympatric host plants. We have previously found that, in general, the damage levels of *A. asclepiadis* are higher on plants with higher levels of lipophilic compounds in the field [25]. As the performance of the herbivores from the Jurmo population was not affected by the content of total lipophilic compounds irrespective of plant origin, it seems probable that the ability to tolerate or detoxify specific lipophilic compounds varies among herbivore populations.

We found that total flavonoids in the leaves had no effect on the performance of the locally adapted herbivores originating from the Anskär population. In contrast, the herbivores form the Jurmo population were negatively affected by an increase in total flavonoids when fed on plants from the Anskär population while flavonoid content had little impact on the Jurmo herbivores when fed on their sympatric host plants or plants from Lammasluoto. It appears that the significant quantitative and qualitative differences in the amounts of flavonoids among the populations [this study and A. Muola, unpublished data] influence the herbivore populations differently. However, based on the current results, flavonoids seem not to influence local adaptation of *A. asclepiadis* as strongly as the lipophilic compounds. We have previously found that the larvae of *A. asclepiadis* prefer plants with higher concentrations of antofine alkaloids [29]. Nevertheless, in this experiment pupal mass of *A. asclepiadis* was not associated with the content of antofine, which is traditionally considered as the most specific toxin in *V. hirundinaria*. However, it is possible that antofine affects other life-history traits than pupal mass, for instance later survival or reproductive success. To conclude, our results suggest that the occurrence and degree of local adaptation of this leaf-feeding herbivore might be modified both by the quantitative and qualitative composition of secondary chemicals of its host plant.

Our results need to be interpreted with caution as there are a myriad of different chemicals involved and the changes in the concentrations of certain chemicals often affect the concentrations of others [52]. Therefore, correlations of herbivore performance and concentration of a particular compound may not necessarily arise from a clear causal relationship. It might be the proportions or the “cocktail” of the different chemicals that are relevant for performance and local adaptation of *A. asclepiadis*. In addition, it is important to be aware of the possibility that some of the significant results might have arisen by chance as multiple separate analyses were conducted.

We carried out our experiment with field collected adult plants and therefore, in addition to genetic differences, the study plants may differ due to environmental factors the plants have experienced in their populations of origin. Thus, all of the variation in plant quality may not be genetically based. However, we have previously shown that there is genetic variation in resistance of *V. hirundinaria* both within and among populations [25], [53]. In addition, herbivore resistance of *V. hirundinaria* is not induced by previous herbivore damage but is more constitutive in nature [A. Muola, unpublished data]. Therefore, the observed differences are likely to be due to genetic differences, as the effects of environmental factors were also minimized by growing the field-collected plants in a common greenhouse environment.

### Conclusions

Here we demonstrate how the occurrence and degree of local adaptation can vary among populations and correlate with qualitative and quantitative variation in plant chemistry among host plant populations. Taken together, our results illustrate how spatial variation in specific defensive traits may drive local adaptation of a potentially coevolving plant-herbivore interaction. Identifying the mechanisms that drive geographic variation in local adaptation is an important starting point in understanding the dynamics of the evolution and coevolution of interacting species.

## Supporting Information

Table S1 Results of ANCOVA-model on the effects of plant and herbivore population of origin, herbivore sex, and leaf chemistry on pupal mass of *Abrostola asclepiadis*. We had three herbivore and three plant populations in a reciprocal feeding trial. Interactions between plant population, herbivore population and five chemical compounds are included in this ANCOVA-model.
(DOCX)
