# Supplementary material for: Plant Chemistry and Local Adaptation of a Specialized Folivore
Source: PLoS One. 2012 May 30;7(5):e38225. doi: 10.1371/journal.pone.0038225 (PMC3364215; doi:10.1371/journal.pone.0038225)
Supplement: Table S1 — Results of ANCOVA-model on the effects of plant and herbivore population of origin, herbivore sex, and leaf chemistry on pupal mass of Abrostola asclepiadis. We had three herbivore and three plant populations in a reciprocal feeding trial. Interactions between plant population, herbivore population and five chemical compounds are included in this ANCOVA-model. (DOCX) [file pone.0038225.s001.docx]

**Table S1.** **Results of ANCOVA-model on the effects of plant and herbivore population of origin, herbivore sex, and leaf chemistry on pupal mass of *Abrostola asclepiadis*.** We had three herbivore and three plant populations in a reciprocal feeding trial. Interactions between plant population, herbivore population and five chemical compounds are included in this ANCOVA-model.

| **Source of variation** | **df** | **F** | ***p*** |
| --- | --- | --- | --- |
| Plant population | 2 | 2.81 | 0.077 |
| Herbivore population | 1 | 0.25 | 0.622 |
| Sex | 1 | 3.82 | 0.061 |
| Plant population × Herbivore population | 2 | 5.13 | 0.013 |
| Plant population × Sex | 2 | 1.25 | 0.301 |
| Herbivore population × Sex | 2 | 2.60 | 0.092 |
| Plant population × Herbivore population × Sex | 2 | 0.19 | 0.825 |
| Lipophilic compounds | 1 | 1.61 | 0.215 |
| Flavonoids | 1 | 3.88 | 0.059 |
| Chlorogenic acid | 1 | 2.32 | 0.139 |
| Catechin derivatives | 1 | 0.78 | 0.384 |
| Antofine | 1 | 4.23 | 0.049 |
| Plant population × Lipophilic compounds | 2 | 4.06 | 0.028 |
| Plant population × Flavonoids | 2 | 0.80 | 0.461 |
| Plant population × Chlorogenic acid | 2 | 1.62 | 0.216 |
| Plant population × Catechin derivatives | 2 | 2.67 | 0.087 |
| Plant population × Antofine | 2 | 1.30 | 0.288 |
| Herbivore population × Lipophilic compounds | 1 | 0.08 | 0.774 |
| Herbivore population × Flavonoids | 1 | 0.85 | 0.365 |
| Herbivore population × Chlorogenic acid | 1 | 1.02 | 0.322 |
| Herbivore population × Catechin derivatives | 1 | 0.01 | 0.915 |
| Herbivore population × Antofine | 1 | 0.76 | 0.391 |
| Plant popul. × Herbivore popul. × Lipophilic compounds | 2 | 3.16 | 0.058 |
| Plant popul. × Herbivore popul. × Flavonoids | 2 | 3.88 | 0.033 |
| Plant popul. × Herbivore popul. × Chlorogenic acid | 2 | 0.03 | 0.970 |
| Plant popul. × Herbivore popul. × Catechin derivatives | 2 | 0.56 | 0.580 |
| Plant popul. × Herbivore popul. × Antofine | 2 | 0.59 | 0.560 |
| Error | 28 |  |  |
